# Reciprocal Prospective Relationships Between Loneliness and Weight Status in Late Childhood and Early Adolescence

**DOI:** 10.1007/s10964-018-0867-9

**Published:** 2018-05-28

**Authors:** Pamela Qualter, Ruth Hurley, Alice Eccles, Janice Abbott, Michel Boivin, Richard Tremblay

**Affiliations:** 1 Manchester Institute of Education, University of Manchester, Oxford Road, Manchester, M13 9PL UK; 2 The School of Psychology, The University of Central Lancashire, Preston, UK; 3 Université Laval, Québec, QC G1V 0A6 Canada; 4 The University of Montreal, Québec, Canada

**Keywords:** Loneliness, Body mass index, Coping, Income sufficiency, Socioeconomic status, Obesity

## Abstract

Adolescents who do not conform to weight ideals are vulnerable to disapproval and victimization from peers in school. But, missing from the literature is a prospective examination of weight status and feelings of loneliness that might come from those experiences. Using data from the Québec Longitudinal Study of Child Development, we filled that gap by examining the prospective associations between loneliness and weight status when the sample was aged 10–13 years. At ages 10, 12, and 13 years, 1042 youth (572 females; 92% from French speaking homes) reported on their loneliness and were weighed and measured. Family income sufficiency was included in our analyses given its relationship with weight status, but also its possible link with loneliness during early adolescence. The findings showed that (1) weight status and loneliness were not associated concurrently; (2) weight status predicted increases in loneliness from ages 12 to 13 years; and (3) loneliness predicted increases in weight from ages 12 to 13 years among female adolescents, but weight loss among male adolescents. The fact that loneliness was involved in weight gain for females suggests that interventions focused on reducing loneliness and increasing connection with peers during early adolescence could help in reducing obesity.

## Introduction

Social relationships and weight are prevalent concerns during adolescence (Denneel et al. 2018; Markey 2010). Adolescents are vulnerable to peer disapproval of body size (Lawler and Nixon 2011), feelings of loneliness (Qualter et al. 2015) and they are driven by a need to “fit in” (Reitz et al. 2014). Given the stigma associated with being overweight or obese, peer disapproval is high, creating negative social consequences (Harrist et al. 2016; Puhl et al. 2016), heightened loneliness among 10–14 years olds (Hayden-Wade et al. 2005) and weight concerns in pre-adolescents (Sinton et al. 2012). However, the prospective relationship between obesity and loneliness has yet to be examined.

Building on longitudinal work that not only indicates that obesity predicts depression, but that depression also predicts obesity (Goldschmidt et al. 2010), we hypothesized a bidirectional relationship between loneliness and high weight status. Conversely, feelings of loneliness are reported by those with eating disorders characterized by low body weight (Puhl and Suh 2015) and a meta-analysis indicated that low weight and depressive symptoms are bidirectionally-related over time (Puccio et al. 2016). Therefore, the current work with adolescents prospectively examines the relationship between (a) loneliness and high weight status and (b) loneliness and low body weight.

### The Social Context of Weight Status

The bio-ecological framework highlights two contexts important for adolescent health because they influence beliefs and behavior: (1) the immediate peer group, and (2) the wider social context within which the individual and peers live (Bronfenbrenner 2005). Social exclusion by peers in school and the accompanying feelings of loneliness during adolescence are recognized as significant influences of adolescent health (Hawkley and Capitanio 2015). But, social exclusion based on weight status needs to also be understood within the wider social context, where social norms of the ideal body size create stigma associated with non-ideal weight, influencing adolescents to criticize their peers’ appearance (Lawler and Nixon 2011) and tease them for non-conformation to weight ideals (Mooney et al. 2009).

While male and female adolescents are often criticized about their appearance by their peers to a similar degree (Lawler and Nixon 2011), the social norms of ideal weight are different for males and females because gender stereotypes of the socio-cultural ideal of beauty emphasize thinness for women and female adolescents (Puhl and Brownell 2001). Such findings suggest that female adolescents may be more vulnerable to the social context of appearance than male adolescents, receiving greater pressure from peers to conform to the socio-cultural ideals. Empirical evidence shows that females are particularly vulnerable to the negative social effects of high weight status, experiencing more rejection and victimization from the peer group than boys after the age of seven years (Qualter et al. 2015). Thus, gender-based social norms are important to consider when examining the interaction between weight status and loneliness because females may be particularly vulnerable to the negative social effects of not conforming to weight status ideals, experiencing, as a result, more loneliness than boys.

Other society level influences are also important to consider in the current work. Socioeconomic adversity during childhood and adolescence is linked to developmental processes: low income is a known risk factor for obesity earlier in development (Grow et al. 2010) because it leads to stress responses that exacerbate metabolic processes, leading to increased weight status (Wickrama et al. 2013). Further, there is reason to think that low income may also be linked to loneliness. To our knowledge, there is currently no research on socioeconomic status and loneliness during adolescence, but parents who have limited resources and income may not find adequate time to spend with their children, contributing to increasing distance between parents and their children and child negligence; they also may not have the financial resources to ensure their child’s engagement in specific peer group activities that cost money. For those reasons, we include family income sufficiency in our analyses.

### Weight Status and Depressive Symptoms

Being at the extremes of weight status (overweight/obese or underweight) is associated with depressive symptoms (Rankin et al. 2016; Puccio et al. 2016). Findings show that obese 12–14 year olds have increased chance of developing depression (Eschenbeck et al. 2009), but also that depressive symptoms predict weight gain (Goldschmidt et al. 2010). The same bi-directional effects have been found for those with low weight status too, with low weight predicting depressive symptoms, but also depressive symptoms predicting decreases in weight (Puccio et al. 2016). The effects appear to be more pronounced for adolescent girls compared to boys (Anderson et al. 2006).

Mechanisms linking depression with obesity (Goldschmidt et al. 2017) and low weight (Puccio et al. 2016) during adolescents have been examined. That evidence shows that distress caused from the feelings of shame and guilt of not conforming to the body and weight ideal contribute to obesity and low weight being linked over time with depression. Goldschmidt et al. also showed that depressive symptoms predict engagement in emotional eating as a way to alleviate distress.

### Weight Status and Loneliness

In contrast to the work exploring weight status and depressive symptoms during adolescence, there is a paucity of research examining concurrent and prospective relationships between weight status and loneliness at that same development period. Loneliness is the negative feeling that occurs when a person does not perceive their social relationships to be as satisfying as they would like (Perlman and Peplau 1981), sharing one common symptom with depression (negative affect). But, loneliness is determined, specifically, by a negative emotional response to a lack of close affiliative ties to peers during adolescence; depression, in contrast, is attributed to a broader range of causes, including determinants other than deficient social relations (i.e., the individual’s genetic makeup, neurological disorders, psychological dysfunctions; Koenig and Abrams 1999). Thus, an exploration of the prospective association between weight status and loneliness is important given that loneliness specifically taps distress associated with peer group problems, which are hypothesized to be a significant part of the puzzle linking weight status with depression.

Research exploring the mechanisms linking weight status and depression discusses (1) how peer problems are a catalyst for depressive symptoms among adolescents with the highest and lowest weight statuses, but also (2) how coping with emotional distress can lead to emotional eating and increases in weight status. Based on the fact that loneliness includes the same negative affect as depression, we hypothesize a prospective bidirectional association between loneliness and weight status, which we discuss further below.

### Weight Status Predicting Loneliness

It is clear, from empirical research, that there is stigma attached to high weight status. Empirical evidence shows that, in western society, the high degree of social stigma attached to obesity/overweight is evident as early as pre-school (Turnbull et al. 2000), with increasing negative ratings for “chubby” body types by children aged 5–10 years (Brylinsky and Moore 1994) and peer social rejection of obese children at ages 6–7 years (Harrist et al. 2016). Those negative social circumstances continue into adolescence, with obese and overweight adolescents being regularly stigmatized, socially excluded, and victimized by peers (Puhl and Luedicke 2012; Puhl et al. 2016). Thus, high weight status is associated with poorer peer relationships, with overweight 10–14 year olds reporting higher levels of loneliness (Hayden-Wade et al. 2005). Given that evidence, one could posit that higher weight status would result in less satisfaction with social relationships over time i.e., greater feelings of perceived loneliness. To date, however, the prospective link between higher weight status and feelings of loneliness has not been examined.

In addition to predicting that higher weight status could result in greater loneliness, it is also possible that those with low weight status will report increasing loneliness over time. Empirical work by Wang et al. (2010) shows that male and female adolescents with high weight status are often bullied by their peers, but underweight girls are also often victims. That aggression, and the accompanying feelings of shame and social isolation from peers, is thought to be a consequence of intrasexual competition that is promoted by society’s emphasis on thinness among females (Vaillancourt 2013). Rotenberg et al. (2013) and Rotenberg and Sangha (2015) have also shown that adolescents with low weight status as a result of eating pathologies report higher levels of loneliness compared to their peers, a consequence of stigmatization and alienated from the peer group.

Given the work detailed above, there is a need to examine whether both high and low weight status predict increasing loneliness over the adolescent years. We postulate that there will be concurrent and prospective associations between weight status and loneliness, and that those relationships will be curvilinear in nature, such that individuals with either the highest or lowest weight statuses will feel more loneliness during adolescence compared to their normal weight peers. The current study will examine the prospective curvilinear associations between weight status and loneliness.

### Loneliness as a Predictor of Increasing Weight Status

Among adults, loneliness has been tentatively implicated as a risk factor for increased weight, but there are few empirical studies. Consistent with the affect-regulation model of binge-eating (Heatherton and Baumeister 1991), evidence shows that induced loneliness leads to increased eating as a way to alleviate distress (Rotenberg and Flood 1999). But, loneliness has also been shown to increase stress hormones such as cortisol that come from heightened physiological stress during episodes of loneliness (Cacioppo and Hawkley 2003), and those stress hormones affect fat storage and transportation with the body (Dallman 2010; Moyer et al. 1994).

In addition to the direct weight-related effects of stress hormones, a combination of perceived stress, disturbed sleep, and cognitive rumination resulting from loneliness (Zawadzki et al. 2013) could affect eating behavior by impairing one’s ability to address problems, leading to greater use of passive coping strategies, such as emotional eating (Nolen-Hoeksema et al. 2008). Those loneliness-related stressors are also argued to increase our seeking of easy learned-rewards such as high fat and high sugar foods due to stimulation of the motivation and reward circuits in the brain (Dallman 2010; Hanlon et al. 2016), making it easier to gain weight and harder to lose it.

The work represented above supports the idea that loneliness may lead to increases in weight status, but the prospective link has not yet been examined. The current study examines whether loneliness predicts increases in weight during late childhood/early adolescence.

## The Current Study

An examination of the reciprocal prospective relationships between loneliness and weight status among adolescence is important if we want to offer effective intervention solutions for loneliness and obesity among youth. In the current study, feelings of loneliness and weight status are explored in a population sample of Canadian 10–13 year olds, and we examine whether high and low weight status put adolescents at risk of later loneliness, but also whether loneliness scores predict increasing weight status. The findings could have important implications for interventions for obese or underweight youth, and for adolescents who report loneliness. Guided by the bioecological framework, we also seek to examine gender and income sufficiency as moderators of the prospective relationships between weight status and loneliness.

## Method

This study utilized data from the Québec Longitudinal Study of Child Development (QLSCD), a large on-going study which has tracked the health and wellbeing of a random sample of Quebec infants on a range of measures since they were 5 months old (see the study website http://www.iamillbe.stat.gouv.qc.ca/default_an.htm for further details). The representative sample is made up of children born throughout 1998 in the Canadian province of Québec (total population over 7 million, with approximately 70,000 newborns per year). A total of 2,940 infants were selected for QLSCD through a region-based stratified sampling design, of which 2120 infants (48.8% girls) took part, with parents providing informed consent in 1998. Twins and children with major diseases at birth were not part of the study. In the current study, four waves of data were used from successive data collections when the sample of children was aged 10, 12, and 13 years. We refer to these time points as T1, T2, and T3 respectively of the current study. The QLSCD was approved by the Health Research Ethics Committees of the Québec Statistics Institute and the University of Montreal.

### Participants

Loneliness, weight, and height data were collected from 1259 children/early adolescents (667 females, 592 males) at T1–T3 of the current study. Participants with and without all data for the period of the current study were compared using Little’s missing completely at random test (Little 1988) to determine whether data imputation would be possible. That comparison resulted in a significant chi-square value, Χ^2^ (18) = 35.246, *p* = .009, suggesting that missing values could not be dealt with using data imputation methods. Thus, we used listwise deletion of cases, analyzing data from 1042 participants (572 females, 470 males) for whom complete loneliness, height, and weight data were available at all three time points^1^. Table 1 shows how the final sample in the current study compares to the original QLSCD sample that had been chosen as a representative sample of children in Québec in 1998. The table shows that, while there were fewer males in the current sample than in the original sample, the reduced sample taking part in the current study was representative of the same children living in the province of Québec in 1998.

**Table 1:** Characteristics of the Sample at the Start of the Quebec Longitudinal Study of Child Development (QLSCD) survey and at T1–T3 of the current study

Time point	Age of participants in months (SD)	n =
Start of QLSCD survey in 1998	4.5 (0.55)	2120
T1 current study	121.70 (3.10)	1042
T2 current study	145.60 (3.05)	1042
T3 current study	157.60 (3.12)	1042

Demographics taken at start of QLSCD for the current sample compared to original sample		
	Sample chosen for QLSCD	Sample in current study
Males (%)	51	45
French-speaking families (%)	81	92
Mother’s age in years	29	31
Father’s age in years	31	33
Mothers did not hold a high school degree (%)	17	15
Fathers did not hold a high school degree (%)	20	16
Mother had a university degree (%)	28	32
Father had a university degree (%)	26	27
Income Sufficiency at normal levels^a^	81	88
Households headed by single parent (%)	7	5

^a^Statistics Canada’s low-income cut-offs (LICOs) were used to categorize the families of participants on income sufficiency. LICOs are income thresholds at which a family would typically spend 20% more of its income than the average family on the necessities of food, shelter, and clothing. Families are classified as having “sufficient income” when the household income is above the low-income threshold determined by Statistics Canada in any given year. When income is between 60 and 90% of the low-income threshold, households are classified as having “insufficient income”; income levels below 60% of the low-income threshold are considered as “very insufficient”

*QLSCD* Quebec longitudinal study of child development

### Measures

#### Loneliness

Loneliness was measured using the Loneliness and Social Satisfaction questionnaire developed by Rotenberg et al. (2004). This three-item measure is similar to the 3-item short form of the UCLA (University of California, Los Angeles) Loneliness Scale (Hughes et al. 2004), but the word “isolated” was simplified to “alone” in item 3. Items asked the extent to which, in the last two weeks, participants had felt (1) “they had people they could talk to”, (2) “left out of things” and, (3) “alone”. Item 1 was reverse coded so that higher scores represented higher feeling of loneliness. Participants answered how they best described those feelings (1 = never, 2 = sometimes, 3 = always), with possible total scores ranging from 3 to 9. Cronbach’s alpha for the loneliness measure was .66, .68, and .74 at T1, T2, and T3 respectively. Total scores on the loneliness scale were used in our regression analyses looking at the prospective associations between loneliness and weight status, but we also created loneliness groups to examine whether children who reported higher levels of loneliness at any given time also had higher weight status scores (higher BMI) at that same time point. Following Yang and Victor (2011) we created a “frequently lonely” group of participants who scored 6 or above on the loneliness scale; all remaining youth were grouped into a “not lonely/occasionally lonely” category. Those groups were created for the purpose of making comparisons on BMI at each time point.

#### Weight status

Trained research assistants weighed and measured the participants when they were wearing lightweight clothing and no shoes. Two measurements were taken, and, if they varied by more than 0.5 cm for height or 0.2 kg for weight, a third measurement was taken. Where multiple measures were available, we used the average of each measure to calculate BMI (BMI = kg/m2). The computation of the participants’ BMI was followed by the creation of a BMI z-score using respondents’ BMI, self-reported age, gender, and the external reference sample from WHO (Cole et al. 2000; de Onis et al. 2007). Those BMI z-scores (referred to here as z-BMI) were used in our regression analyses, exploring the prospective association between weight status (z-BMI) and loneliness. As well as create a z-BMI score for each participant, we also classified each of them as underweight, overweight, obese, or normal weight, following recommendations from The International Obesity Task Force BMI (IOTF; Cole et al. 2007). Those recommendations classified participants into the following weight categories at each time point: thin grade 3 (BMI ≤ 16), thin grade 2 (BMI ≤ 17 & > 16), thin grade 1 (BMI ≤ 18.5 & > 17), overweight (BMI ≥ 25 & < 30), obese (BMI ≥ 30) morbid obesity (BMI > 30), and normal weight (BMI ≥ 18.6 & ≤ 24.5). Those IOTF cut-offs are recommended for international research and comparison (Lobstein et al. 2004), and are used here to create weight status groups for each time point, enabling us to explore whether different weight categories reported higher levels of loneliness at each time point.

#### Income sufficiency

Sufficiency of income was determined by low-income cutoffs set by Statistics Canada in any given year. It takes into account household income, the size of the household, and the size of the residence area. Statistics Canada’s low-income cut-offs (LICOs) are income thresholds at which a family would typically spend 20% more of its income than the average family on the necessities of food, shelter, and clothing (https://www.statcan.gc.ca/pub/75f0002m/2012002/lico-sfr-eng.htm). Families are classified as having “sufficient income” when the household income is above the low-income threshold determined by Statistics Canada. When income is between 60 and 90% of the low-income threshold, households are classified as having “insufficient income”; income levels below 60% of the low-income threshold are considered as “very insufficient”. Although LICOs are widely used, they do not measure poverty. Unlike the US, Canada does not have a measure of poverty. For example, according to Statistics Canada (https://www.statcan.gc.ca/pub/75f0002m/75f0002m2010003-eng.htm) low-income thresholds are different for a family living in a rural area compared to similar families living in large cities.

### Analyses Plan

First, using ANOVA, we examined whether there were differences between people in the different weight categories on loneliness at each time point. Second, using T-tests, we examined differences in BMI between groups of youth identified as either “frequently lonely” or “not lonely/occasionally lonely”. We conducted all analyses separately by gender. Third, we conducted a series of chi-square tests to clarify the associations between loneliness and weight status, exploring for males and females separately, membership of any given loneliness group by weight category at each time point. Those chi-square analyses helped us to understand whether adolescents who were “frequently lonely” at any time point were more likely than chance to also be members of the obese or underweight weight groups. Together, those sets of analyses enabled us to examine concurrent relationships between loneliness and weight status.

Fourth, we examined prospective linear and curvilinear relationships between (1) z-BMI and loneliness, and (2) loneliness and z-BMI using Hierarchical Regression Analyses (HRAs), controlling for gender and income sufficiency. Curvilinear analyses were used to determine the exact associations between weight status at one time point and loneliness at the following data collection waves, and loneliness at one time point and weight status at the other data collection waves. Based on our findings of quadratic effects between z-BMI and loneliness, Structural Equation Modelling was not an appropriate analytic tool and the HRAs are presented as our final statistical analyses.

## Results

We examined differences in mean loneliness for young people in different weight categories using ANOVAs, looking at each time point and males and females separately. Because there were so few participants in the three grades of thinness as defined by (Cole et al. 2007), we merged those groups to create one group that we defined as “underweight” (BMI < 18.5). We also merged the obese and morbidly obese groups given that there were so few participants classified as morbidly obese.

The one-way ANOVAs investigating loneliness by weight category revealed no significant differences at any of the three time periods: T1 (age 10 years) females, F (3, 568) = .994, *p* = .395, males, F (3, 466) = 1.057, *p* = .367; T2 (age 12 years) females, F (3, 568) = .741, *p* = .528, males, F (3, 466) = 1.835, *p* = .140; and T3 (age 13 years) females, F (3, 568) = .014, *p* = .998, and males, F (3, 466) = .393, *p* = .758. Thus, in the current population sample, it seems weight status is not associated with concurrent reports of higher loneliness during adolescence for males or females; those with high or low weight status did not report higher levels of loneliness compared to normal weight category to report feelings of loneliness (Table 2).

**Table 2:** Mean (and standard deviations) for loneliness in each weight category at each time point for females and males

Time (age)	Underweight BMI < 18.5		Normal range 18.51–24.99		Overweight BMI ≥ 25		Obese BMI ≥ 30	
	Females	Males	Females	Males	Females	Males	Females	Males
T1 (10 year)	3.64 (.93)	43.46 (.95)	3.82 (1.19)	3.88 (1.23)	3.97 (1.33)	3.86 (1.29)	4.03 (1.27)	3.72 (.96)
*N* = 1042	36	26	407	325	99	90	30	29
T2 (12 year)	3.71 (.86)	3.28 (.61)	3.76 (1.19)	3.81 (1.25)	3.90 (1.17)	3.73 (1.09)	3.97 (1.17)	3.58 (.97)
*N* = 1042	35	25	384	311	116	98	37	36
T3 (13 year)	3.80 (1.10)	3.93 (1.51)	3.83 (1.31)	3.92 (1.29)	3.85 (1.14)	4.06 (1.43)	3.84 (1.26)	3.82 (1.29)
*N* = 1042	30	28	384	299	120	105	38	38

*Notes*: Sample participants were categorized using the following recommendations from The International Obesity Task Force BMI (IOTF; Cole et al. 2007): thin grade III (BMI ≤ 16), thin grade II (BMI ≤ 17 & > 16), thin grade I (BMI ≤ 18.5 & > 17), overweight (BMI ≥ 25 & < 30), obese (BMI ≥ 30), morbid obesity (BMI > 30), and normal weight (BMI ≥ 18.6 & < 24.5). Underweight = Grade I, II, III were combined due to low numbers in Grades II and III; Obese (obese and morbid obese were combined due to low numbers in the morbid obese category); Possible loneliness scores ranged from 3 to 9. *N* = 1042 (Female = 572; Male = 470). ANOVAs revealed no differences between males and females on feelings of loneliness at each time point

The next set of analyses examined whether adolescents classified as “frequently lonely” were different to peers categorized as “not lonely/sometimes lonely” on weight status at each of the time points. We ran a series of independent t-tests, separately for males and females and found no difference between the “frequently lonely” and “not lonely/sometimes lonely” groups at age 10 years (T1: females, t (74.78) = 1.88, *p* = .064; males, t (465) = 1.12, *p* = .264) or age 12 years (T2: females, t (570) = 1.26, *p* = .210; males, t (467) = .03, *p* = .974). At age 13 years (T3), there was a difference between the lonely groups for males (t (81.38) = 3.04, *p* = .003), but not females (t (77.77) = 1.53, *p* = .131). Findings showed that male adolescents aged 13 years in the “frequently lonely” group had a higher weight status than their male peers in the “not lonely/sometimes lonely” group (Table 3).

**Table 3:** Mean (and standard deviations) for BMI by loneliness group at each time point for females and males

Time	Frequently lonely		Not lonely/Sometimes lonely	
	Females	Males	Females	Males
T1 (10 year)	19.26 (4.17)	17.76 (2.32)	18.28 (3.11)	18.25 (2.06)
N = 1042	66	54	506	416
T2 (12 year)	20.63 (3.43)	19.86 (3.92)	19.98 (3.88)	19.84 (3.76)
N = 1042	60	49	512	421
T3 (13 year)	21.82 (5.39)	22.52 (5.11)	20.80 (3.84)	20.56 (3.79)
N = 1042	69	69	503	401

*Notes*: Those in the “Frequently Lonely” group scored 6 or above on the loneliness scale; those scoring between 3 and 5 were classified as “Not Lonely/Sometimes Lonely”; N = 1042 (Female = 572; Male = 470); T-tests showed no differences on BMI between same sex peers in the “Frequently Lonely” and “Not Lonely/Sometimes Lonely” groups at ages 10 and 12 years. At age 13 years, males in the “Frequently Lonely” group scored significantly higher on BMI compared to their same sex peers in the “Not Lonely/Sometimes Lonely” group; there were no significant differences between females. All BMI mean scores would be considered in the normal range according to The International Obesity Task Force BMI Cut-offs (IOTF; Cole et al. 2007) where normal weight is considered to be BMI ≥ 18.6 & < 24.5

Given that the mean BMI scores at T3 for males in the “frequently lonely” group would be considered within the normal range of BMI scores according to The International Obesity Task Force BMI cut-offs (Cole et al. 2007; normal weight = BMI ≥ 18.6 & ≤ 24.5), we decided to explore the concurrent association between loneliness and weight status at T3 further. We conducted two Fisher-Freeman-Halton tests, one for males and one for females. Those analyses showed that, at age 13 years (T3), males classified as “frequently lonely” were more likely than chance to be in the normal weight category, while boys in the “not lonely/sometimes” group were more likely to be in the underweight category and less likely than chance to be in be in the normal weight group and overweight weight status groups (z = 7.817, *p* = .012). That was not the case for girls at age 13 years (z = 2.096, *p* = .558). Thus, it seems that the significant difference in BMI scores between the “frequently lonely” versus “not lonely/sometimes lonely” groups of 13-year old males was driven by the higher numbers of males from the “not lonely/sometimes lonely group” in the underweight category than we would expect by chance. Taken together with the results from the ANOVA, t-tests, and chi-square analyses show no concurrent relationships between the highest and lowest weight status and loneliness.

To examine prospective effects of weight status on loneliness (DV) we ran a series of Hierarchical Multiple Regressions, with predictors on the following steps: (1) gender, income sufficiency, and loneliness (HRAs) from the earlier time point, (2) z-BMI from the earlier time point, (3) z-BMI squared (z-BMI^2^), (4) z-BMI x gender interaction, and (5) z-BMI^2^ x gender interaction. The squared (^2^) term serves as a test for a quadratic relation (Cohen et al. 2003). Scores were centered using grand mean subtraction for loneliness, and BMI z-scores. Gender and income sufficiency were dummy coded (gender: −1 = female, and + 1 = male; + 1 = income sufficiency, and −1 = insufficient, with the latter including the categories of insufficient and very insufficient) as recommended by Cohen et al. (2003). We performed bootstrapping, estimating a 95% bias-corrected confidence interval for all values of interest (1000 bootstrap sample).

A further set of HRAs was conducted to examine the longitudinal over-time effects of loneliness on BMI. The regressions followed the pattern and procedure outlined above, with z-BMI at each time point as the independent variable, and loneliness and z-BMI from earlier time points as predictors. Any two-way interactions between z-BMI^2^ x Gender were further examined by testing for the linear and quadratic (curvilinear) relations on the measure for each gender separately. Key information from the HRAs are detailed in the manuscript text, with tables (Tables S1-S6) detailing all HRA information included as on-line supplementary information.

### Stability of Weight and Loneliness Over Time

As anticipated, over time the strongest predictor of z-BMI and loneliness were previous measures of the same construct, confirming stability. The strongest effects for both constructs were seen over the one-year interval between age 12 and 13 years (T2 to T3). z-BMI stability co-efficients ranged from β = .89 (SE = .02) to .95 (SE = .02), with the highest stability being one year to the next. Loneliness beta weights ranged from β = .27 (SE = .03) to .47 (SE = .04), with moderate to large effects (per Cohen 1988).

### Effects of Income Sufficiency on Weight Status and Loneliness

Bootstrapped findings revealed no influence of earlier income sufficiency on weight status (β = .00 [SE = .02] to −.02 [SE = .02]) or loneliness (β = .05 [SE = .06] to −.11 [SE = .07]).

### Weight Status Predicting Loneliness

**Fig. 1:**
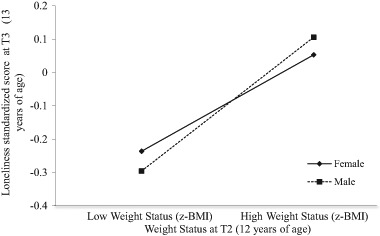
Weight Status at T2 (age 12 years) and Loneliness at T3 (13 years)

Bootstrapped findings showed only one significant linear effect between weight status at T2 and loneliness at T3 (β = .11 [SE = .04], *p* = .007), suggesting that higher weight status at age 12 predicted higher loneliness at age 13 years. That effect is detailed in Fig. 1. There were no other significant linear or quadratic effects of z-BMI on loneliness over time (See Tables S1–3 for details).

### Loneliness Predicting Weight Status

**Fig. 2:**
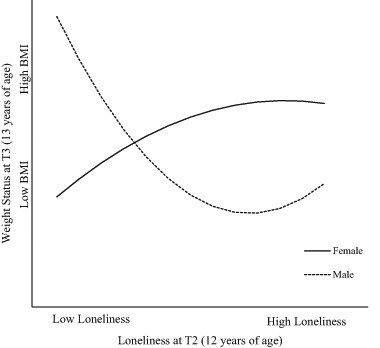
Slopes of the relation between loneliness T2 (age 12 years) and BMI T3 (age 13 years) as a function of gender

Bootstrapped findings showed a quadratic effect of loneliness at T2 (age 12 years) that interacted significantly with gender to predict z-BMI at T3 (age 13 years), β = .02, SE = .01, *p* = .007 (see Table S6 for full results). Figure 2 illustrates the quadratic gender-mediated effect, showing that (a) higher loneliness at age 12 years was related to higher z-BMI at age 13 years for girls, but (b) high loneliness at age 12 years was related to lower z-BMI at age 13 years for boys. This result suggests that loneliness may have a particular role in increasing weight for girls and reducing weight for boys. There were no other significant linear or quadratic effects of loneliness on z-BMI over time (See Tables S4–6 for details).

## Discussion

The relationships between weight status and loneliness are complex. Weight status was not associated with concurrent feelings of loneliness, but higher weight at age 12 years predicted increased loneliness from age 12 to 13 years. Interestingly, for those who reported loneliness at age 12 years, we observed a differential effect: weight gain for girls and weight loss for boys. That is likely to have detrimental psychosocial consequences for both girls and boys given that the perceived ideal female body is slim, but the ideal male body shape is muscular (Field et al. 2014). Findings also suggest a significant health consequence for girls that are lonely, with increases in weight, which for girls already overweight or obese, is a significant health risk.

### The Effect of Weight Status on Loneliness

Due to the increasing importance of peer acceptance and body image concerns during early adolescence (Denneel et al. 2018; Markey 2010), and the specific peer problems encountered by youth at the extremes of weight status (Puhl et al. 2016), we investigated whether extremes of weight status would predict greater loneliness. Unlike previous research (Hayden-Wade et al. 2005), we did not find concurrent associations between high weight status and loneliness. We did find prospective associations between high weight status and loneliness, with male and female adolescents with higher weight status at age 12 years reporting increased loneliness between 12 and 13 years. Such findings that there were no within-time gender differences are consistent with previous research showing that male and female adolescents experience weight related criticism to a similar degree (Lawler and Nixon 2011). Further, the fact that those with higher weight status reported increasing loneliness highlights the fact that the peer context is important for understanding the social norms surrounding weight status, with society’s body ideals communicated and reinforced by peers (Jones and Crawford 2006); the fact that the prospective effects linking high weight status and loneliness are only evident at ages 12 to13 years and not 10–12 years suggests a sensitive period in development when the peer context becomes particularly influential in delivering messages about ideal body size.

Future research will want to examine whether weight status exerts effects on loneliness only under specific circumstances, such as those where there is a high-level of weight-based victimization (Juvonen et al. 2017) or an internalization of the appearance ideals (Lawler and Nixon 2011), or when internalizing problems, such as depression, already exist. How loneliness explicitly links to weight-based victimization and self-esteem should also be examined. In addition, time-specific influences of the peer context – in and outside school- that explain the link between high weight status and loneliness should be examined to determine whether there are sensitive periods during which adaptive responses to peer relationship difficulties can be most effective.

Contrary to expectations, in our study low weight was not found to be concurrently associated with, or a significant predictor of, loneliness. The number of participants who were classified as underweight was small, making it more difficult to gauge those effects. That said, it is also possible that having low weight status is less socially stigmatizing than being overweight or obese. It is possible that, due to differences in assumptions made about the volitional cause of high weight status (Puhl and Heuer 2010), it is the case that those who are underweight have fewer social problems. Underweight could also be easier to disguise than overweight, leading to fewer negative social repercussions.

### The Effect of Loneliness on Weight Status

In line with predictions, loneliness had a significant prospective impact on weight status interacting significantly with gender at age 12 years to predict weight status at age 13 years. Specifically, female adolescents with higher loneliness at age 12 years gained weight from age 12 to 13 years, while male adolescents with higher loneliness lost weight. Those results contribute to a growing body of research indicating that loneliness affects health (Hawkley and Capitanio 2015), and are consistent with findings that higher loneliness leads to increased food consumption among female older adolescents (Rotenberg and Flood 1999). While we have not examined mechanisms linking loneliness and weight status, we have provided the first evidence that loneliness is directly related to increasing weight for females upon entry into early adolescence.

The finding that loneliness reduces weight for male adolescents might suggest that loneliness serves as an inhibitor of food consumption among those male adolescents; in contrast, loneliness may have disinhibited food consumption for the female adolescents, a significant problem for those girls already overweight or obese females. The reduction in weight by male adolescents with high weight status in the current sample may be demonstrable of a heightened awareness that they had to lose weight to improve their social connections or were simply more motivated to do so. But, it may be the case that boys in the current sample simply dealt with the stigmatization of overweight by inhibiting eating as a way to copy with negative emotions. It is also possible that boys do not experience psychosocial effects until norms around their ideal male body shape (that of muscle; Field et al. 2014) materialize during puberty. Future research should examine those effects.

The weight gains we saw in girls are likely to be the outcome of engagement in emotional eating, a maladaptive method of alleviating negative emotions (Haedt-Matt and Keel 2011), that they used to cope with feelings of loneliness. Goldschmidt et al. (2017) found that poor emotional awareness and limited access to adaptive emotion regulation strategies contribute to emotional eating in adolescent females, so further research will want to establish whether emotional eating and poor emotion regulation can help explain the prospective association between loneliness and weight gain for female adolescents.

The recommended next stage of empirical study is the examination of mechanisms that explain how loneliness increases weight status among adolescent females and decreases weight status for adolescent males. The work detailed above may suggest that male adolescents are more motivated to lose weight or more aware that doing so would lead to increased social acceptance needs to be explored, but the possibility that female adolescents were engaged in emotional eating also needs to be examined. Given that among females, loneliness is associated with ruminative cognitions (Vanhalst et al. 2012), it is possible that rumination directly affects weight by influencing coping strategies (i.e., emotional eating) and planning (i.e., it reduces one’s ability to stick to healthy eating intentions). Future work should examine emotion regulation strategies, including rumination, and explore whether alternative, more adaptive strategies for dealing with loneliness might not lead to weight gain. Such work is important for informing interventions focused on reducing loneliness and/or obesity.

Future research will also want to explore how the physiological effects of loneliness could influence eating behavior including increasing the propensity to binge eat palatable foods and reducing one’s ability to track food consumption. Such future work should take into account the moderating effects of sleep and physical activity as those factors affect weight metabolism and have been shown to be deficient in lonely adolescents (Harris et al. 2013; Pels and Kleinert 2016b). The impact of our findings for interventions to prevent obesity is clear – targeting early adolescents, particularly females, who are lonely, could help in the fight against obesity.

### Stability of Loneliness and Weight Status Over Time

Results also support the stability of weight status and loneliness over time. The relative stability of the constructs over time supports previous research (Pryor et al. 2011; van Dulmen and Goossens 2013). The strong stability of weight status and loneliness reflects the difficult task that intervention teams face in order to affect changes in those areas of health. After age 13 years, the stability of weight status is likely to change due to physiological changes that accompany puberty, and future work will want to examine how the onset of puberty impacts the prospective associations between weight status and loneliness, exploring the impact for male and female adolescents separately. Given that for females, advanced pubertal maturation is associated with internalizing problems, explained exclusively in terms of environmental influences (Marceau et al. 2012), we might expect that the prospective association between weight status and loneliness is also moderated by pubertal timing for girls. The time interval between data collection waves in the study varied from one to two years, but the effects were seen in the one-year time interval between age 12 to age 13 (T2 to T3). That age could be a key sensitive period for peer relationship problems to impact weight status, future longitudinal studies may consider one-year time intervals between waves to give a more nuanced picture of magnitude and persistence of effects over time (VanderWeele et al. 2011).

### Income Sufficiency and Effects on Weight Status and Loneliness

In the current study, we explored the impact of income sufficiency on weight status and loneliness. We did not find any effects linking recent social disadvantage, measured here in terms of income sufficiency, to high weight status. That finding is consistent with other research (Lee et al. 2014) that found only poverty exposure prior to 2 years of age had a robust association with adolescent obesity.

To our knowledge, this is the first study to examine the longitudinal associations between income sufficiency and youth loneliness. We thought it might be the case that children whose parents had limited income did not have adequate time to spend with their children or have the resources to support peer engagement activities, and children from those families would experience increasing distance from parents and peers, and, thus, report loneliness. But, we did not find that adolescents whose families had insufficient incomes as defined by Statistics Canada, reported higher rates of loneliness. Further empirical work should examine income sufficiency in relation to loneliness among youth, examining whether that effect is found for children whose focus is more on parents as the main source of support (Csikszentmihalyi and Larson 1984).

### Strengths and Limitations

This study has several strengths, including the large population sample, the robust anthropometric techniques used to collect weight and height data, and the prospective nature of the design. The current study followed children over a 3-year period into early adolescence and allowed confirmation of the temporal relationships between loneliness, weight status, and income sufficiency, and provided exploration of effects for male and female adolescents. The time period provided a good test of the stability of the constructs during a period of developmental transition. The simplicity, longitudinal design, and youth sample in the current study add depth to our understanding of the links between weight status and social problems faced by young people during a key period in development.

The study is not without limitations. The difference in interval lengths between data collection points could have introduced confounds into the study. Equal time intervals between measurement points would be preferable (VanderWeele et al. 2011). Missing cases were dealt with through Listwise deletion because the Data Missing Completely At Random (MCAR) tests proved significant. Our chosen method is a less favored method of data cleansing and could have introduced bias in the sample by altering the standard error estimates for sub samples due to non-random data (Allison 2002). However, the use of bootstrapping helps provide an assurance that the results are not spurious.

### Areas for Further study

Throughout this discussion we have highlighted important areas of future work. We noted the need to examine whether weight status exerts effects on loneliness only under specific circumstances, including situations where there is a high-level weight-based victimization or internalization of appearance ideals. We also noted the need to further explore the gendered responses to loneliness, and how any differences between male and female adolescents are related to future gains or reductions in weight.

In addition, in future studies, several further variables could be controlled, including activity levels and onset of puberty. Ethnicity could not be explored as a moderator in the current study because the sample was not ethnically diverse. In future work, ethnicity should also be explored given evidence that girls with high weight status in certain ethnic groups suffer less from the negative effects of weight stigma than other groups (Mustillo et al. 2013).

## Conclusion

The current study examined the concurrent and prospective reciprocal relationships between weight status and loneliness, controlling for income sufficiency and gender. We found that both male and female adolescents with higher weight status reported increasing loneliness from ages 12 to 13 years, showing that society’s views of what constitutes an body ideal size and shape are communicated and reinforced by peers at this point in development. In addition, loneliness at age 12 years reduced weight for male adolescents from age 12 to 13 years, and increased weight for female adolescents during that same period, suggesting that loneliness may serve as an inhibitor of food consumption among male adolescents, but may disinhibit food consumption for female adolescents, a significant problem for already overweight or obese females. Further work will want to explore the prospective effects further to determine whether the gendered effect is specific to the current sample, and, if not, what that effect tells us about gendered coping and peer group friendships among male and female youth.

The findings suggest early clinical attention to high weight status and loneliness will be important and may have significant effects for adolescent females. Reducing peer-related problems for those with high weight status will help reduce feelings of loneliness, but such problems reflect society’s stigma attached to non-conformation to body ideals, making such social norms hard to change. Thus, it will be more important to help adolescents develop resilient strategies of coping with peer criticism of non-ideal body size, reducing the chance that they will engage in emotional eating or experience social stress, both of which would lead to weight gains.

## Electronic supplementary material

Supplementary Information

## Footnotes

1 Loneliness, weight, and height data were also available for 926 of the 1042 children at a further time point when the sample was aged 15 years. However, the measure of loneliness was limited to a single item, with response items that did not align with that for the earlier loneliness items; height and weight data were self-reported. In addition, there were so few participants who were classified as underweight (N = 20 females; 9 males) or obese (N = 9 females; 5 males) that it would not make the analyses viable.
