# Supplementary material for: Reciprocal Prospective Relationships Between Loneliness and Weight Status in Late Childhood and Early Adolescence
Source: J Youth Adolesc. 2018 May 28;47(7):1385–97. doi: 10.1007/s10964-018-0867-9 (PMC6002443; doi:10.1007/s10964-018-0867-9)
Supplement: Supplementary file 1 — Supplementary Information [file 10964_2018_867_MOESM1_ESM.docx]

Reciprocal Prospective Relationships Between Loneliness and Weight Status in Late Childhood and Early Adolescence

**Supplementary On-line Information**

**HRA Bootstrapped 5-Step Results Tables**

**Table S1.** Bootstrapped Hierarchical Regression Analysis: T1 z-BMI as a Predictor of Dependent Variable T2 Loneliness including quadratic effects and interactions.

|  |  | Coefficients | | |  | Variance explained in each step | | |
| --- | --- | --- | --- | --- | --- | --- | --- | --- |
|  | Predictors | B (SE) | 95% CI | *p* |  | R2 | ∆R2 | *F* Change |
| 1 | GENDER | -.03 (.04) | [-.10, .05] | .398 | i | .09 | .09 | *F*(3,1016)= 31.56, *p*<.001 |
|  | IS_T1 | -.11 (.07) | [-.25, .02] | .079 |  |  |  |  |
|  | T1_Lon | .27 (.04) | [.20, .34] | .001 |  |  |  |  |
| 2 | GENDER | -.03 (.04) | [-.10, .05] | .400 |  | .09 | .00 | *F*(1,1015)= 1.18, *p*=.278 |
|  | IS_T1 | -.11 (.07) | [-.25, .02] | .083 |  |  |  |  |
|  | T1_Lon | .27 (.04) | [.20, .34] | .001 |  |  |  |  |
|  | T1 z-BMI | .04 (.04) | [-.03, .11] | .302 |  |  |  |  |
| 3 | GENDER | -.03 (.04) | [-.10, .05] | .402 |  | .09 | .00 | *F*(1,1014)=.01, *p*=.915 |
|  | IS_T1 | -.11 (.07) | [-.24, .02] | .088 |  |  |  |  |
|  | T1_Lon | .27 (.04) | [.20, .34] | .001 |  |  |  |  |
|  | T1 z-BMI | .04 (.04) | [-.04, .13] | .342 |  |  |  |  |
|  | T1 z-BMI^2^ | .00 (.02) | [-.05, .03] | .896 |  |  |  |  |
| 4 | GENDER | -.03 (.04) | [-.10, .04] | .390 |  | .09 | .00 | *F*(1,1013)= 1.46, *p*=.228 |
|  | IS_T1 | -.11 (.07) | [-.24, .02] | .097 |  |  |  |  |
|  | T1_Lon | .27 (.04) | [.20, .34] | .001 |  |  |  |  |
|  | T1 z-BMI | .04 (.04) | [-.04, .12] | .337 |  |  |  |  |
|  | T1 z-BMI^2^ | .00 (.02) | [-.05, .03] | .805 |  |  |  |  |
|  | T1 z-BMI x Gender | -.04 (.04) | [-.11, .04] | .242 |  |  |  |  |
| 5 | GENDER | -.02 (.04) | [-.10, .07] | .679 |  | .09 | .00 | *F*(1,1012)=.33, *p*=.565 |
|  | IS_T1 | -.11 (.07) | [-.24, .02] | .093 |  |  |  |  |
|  | T1_Lon | .27 (.04) | [.20, .34] | .001 |  |  |  |  |
|  | T1 z-BMI | .05 (.04) | [-.04, .13] | .292 |  |  |  |  |
|  | T1 z-BMI^2^ | -.01 (.03) | [-.06, .04] | .684 |  |  |  |  |
|  | T1 z-BMI x Gender | -.03 (.04) | [-.11, .06] | .521 |  |  |  |  |
|  | T1 z-BMI^2^ x Gender | -.01 (.03) | [-.06, .04] | .604 |  |  |  |  |

*Note.* T1=10 years old, T2=12 years old; z-BMI created using respondents’ BMI, self-reported age, Gender, and the external reference sample from WHO (Cole et al., 2000; de Onis et al., 2007); Lon= mean centred loneliness score; IS = Income Sufficiency; Bootstrap results are based on 1000 bootstrap samples*; Model:* *F*(7,1012)=13.94, *p*<.001

**Table S2.** Bootstrapped Hierarchical Regression Analysis: T1 z-BMI as a Predictor of Dependent Variable T3 Loneliness including quadratic effects and interactions.

|  |  | Coefficients | | |  | Variance explained in each step | | |
| --- | --- | --- | --- | --- | --- | --- | --- | --- |
|  | Predictors | B (SE) | 95% CI | *p* |  | R2 | ∆R2 | *F* Change |
| 1 | GENDER | -.03 (.04) | [-.10, .05] | .458 | i | .08 | .08 | *F*(3,1016)= 29.22, *p*<.001 |
|  | IS_T1 | -.07 (.07) | [-.21, .06] | .340 |  |  |  |  |
|  | T1_Lon | .30 (.03) | [.23, .36] | .001 |  |  |  |  |
| 2 | GENDER | -.03 (.04) | [-.10, .05] | .467 |  | .08 | .00 | *F*(1,1015)= 3.95, *p*=.047 |
|  | IS_T1 | -.06 (.07) | [-.20, .07] | .387 |  |  |  |  |
|  | T1_Lon | .29 (.03) | [.23, .36] | .001 |  |  |  |  |
|  | T1 z-BMI | .08 (.05) | [-.02, .17] | .094 |  |  |  |  |
| 3 | GENDER | -.03 (.04) | [-.10, .05] | .466 |  | .09 | .00 | *F*(1,1014)= 2.85, *p*=.092 |
|  | IS_T1 | -.06 (.07) | [-.20, .07] | .366 |  |  |  |  |
|  | T1_Lon | .29 (.03) | [.23, .36] | .001 |  |  |  |  |
|  | T1 z-BMI | .03 (.05) | [-.08, .12] | .607 |  |  |  |  |
|  | T1 z-BMI^2^ | .03 (.03) | [-.01, .10] | .125 |  |  |  |  |
| 4 | GENDER | -.03 (.04) | [-.10, .05] | .462 |  | .09 | .00 | *F*(1,1013)=.06, *p*=.807 |
|  | IS_T1 | -.06 (.07) | [-.21, .07] | .361 |  |  |  |  |
|  | T1_Lon | .29 (.03) | [.23, .36] | .001 |  |  |  |  |
|  | T1 z-BMI | .03 (.05) | [-.08, .12] | .606 |  |  |  |  |
|  | T1 z-BMI^2^ | .03 (.03) | [-.01, .10] | .131 |  |  |  |  |
|  | T1 BMI x Gender | .01 (.05) | [-.08, .11] | .830 |  |  |  |  |
| 5 | GENDER | .00 (.05) | [-.09, .10] | .995 |  | .09 | .00 | *F*(1,1012) = 1.57 *p*=.211 |
|  | IS_T1 | -.06 (.07) | [-.20, .07] | .356 |  |  |  |  |
|  | T1_Lon | .29 (.03) | [.23, .36] | .001 |  |  |  |  |
|  | T1 z-BMI | .04 (.05) | [-.07, .13] | .436 |  |  |  |  |
|  | T1 z-BMI^2^ | .02 (.04) | [-.04, .11] | .533 |  |  |  |  |
|  | T1 z-BMI x Gender | .05 (.05) | [-.04, .12] | .326 |  |  |  |  |
|  | T1 z-BMI^2^ x Gender | -.03 (.04) | [-.11, .04] | .443 |  |  |  |  |

*Note.* T1=10 years old; T3=13 years old; z-BMI created using respondents’ BMI, self-reported age, Gender, and the external reference sample from WHO (Cole et al., 2000; de Onis et al., 2007); Lon= mean centred loneliness score; IS = Income Sufficiency; Bootstrap results are based on 1000 bootstrap samples*; Model:* *F* (7,1019) = 13.78, *p*<.001

**Table S3.** Bootstrapped Hierarchical Regression Analysis: T2 z-BMI as a Predictor of Dependent Variable T3 Loneliness including quadratic effects and interactions.

|  |  | Coefficients | | |  | Variance explained in each step | | |
| --- | --- | --- | --- | --- | --- | --- | --- | --- |
|  | Predictors | B (SE) | 95% CI | *p* |  | R2 | ∆R2 | *F* Change |
| 1 | GENDER | .00 (.04) | [-.07, 07] | .933 | i | .19 | .19 | *F*(3,1030)=77.90,*p*<.001 |
|  | IS_T2 | .05 (.06) | [-.08, .17] | .443 |  |  |  |  |
|  | T2_Lon | .47 (.04) | [.40, .55] | .001 |  |  |  |  |
| 2 | GENDER | .00 (.04) | [-.07, .07] | .920 |  | .19 | .01 | *F*(1,1029)=9.86,*p*=.002 |
|  | IS_T2 | .06 (.06) | [-.06, .18] | .343 |  |  |  |  |
|  | T2_Lon | .47 (.04) | [.39, .54] | .001 |  |  |  |  |
|  | T2 z-BMI | .11 (.04) | [.03, .19] | .007 |  |  |  |  |
| 3 | GENDER | .00 (.04) | [-.07, .07] | .916 |  | .20 | .00 | *F*(1,1028)=3.78, *p*=.052 |
|  | IS_T2 | .06 (.06) | [-.06, .18] | .300 |  |  |  |  |
|  | T2_Lon | .47 (.04) | [.39, .55] | .001 |  |  |  |  |
|  | T2 z-BMI | .06 (.05) | [-.03, .14] | .195 |  |  |  |  |
|  | T2z-BMI^2^ | .04 (.02) | [-.01, .09] | .091 |  |  |  |  |
| 4 | GENDER | .00 (.04) | [-.07, .07] | .918 |  | .20 | .00 | *F*(1,1027)=.28, *p*=.599 |
|  | IS_T2 | .06 (.06) | [-.05, .18] | .289 |  |  |  |  |
|  | T2_Lon | .47 (.04) | [.39, .55] | .001 |  |  |  |  |
|  | T2 z-BMI | .06 (.05) | [-.03, .14] | .194 |  |  |  |  |
|  | T2 z-BMI^2^ | .04 (.03) | [-.01, .10] | .086 |  |  |  |  |
|  | T2 z-BMI x Gender | .02 (.04) | [-.07, .10] | .647 |  |  |  |  |
| 5 | GENDER | .02 (.05) | [-.07, .11] | .728 |  | .20 | .00 | *F*(1,1026)=.80, *p*=.371 |
|  | IS_T2 | .06 (.06) | [-.05, .18] | .288 |  |  |  |  |
|  | T2_Lon | .47 (.04) | [.39, .54] | .001 |  |  |  |  |
|  | T2 z-BMI | .07 (.05) | [-.03, .15] | .155 |  |  |  |  |
|  | T2 z-BMI^2^ | .03 (.03) | [-.02, .10] | .311 |  |  |  |  |
|  | T2 z-BMI x Gender | .04 (.05) | [-.05, .14] | .343 |  |  |  |  |
|  | T2 z-BMI^2^ x Gender | -.02 (.03) | [-.08, .05] | .527 |  |  |  |  |

*Note.* T2=12 years old; T3=13 years old; z-BMI created using respondents’ BMI, self-reported age, Gender, and the external reference sample from WHO (Cole et al., 2000; de Onis et al., 2007); Lon= mean centred loneliness score; IS = Income Sufficiency; Bootstrap results are based on 1000 bootstrap samples*; Model:* *F* (7,1026)= 35.84, *p*<.001

**Table S4.** Bootstrapped Hierarchical Regression Analysis: T1 Loneliness as a Predictor of Dependent Variable T2 z-BMI including quadratic effects and interactions.

|  | | Coefficients | | |  | Variance explained at each step | | |
| --- | --- | --- | --- | --- | --- | --- | --- | --- |
| Predictors | | B (SE) | 95% CI | *p* |  | R2 | ∆R2 | F Change |
| 1 | GENDER | .00 (.01) | [-.02, .03] | .909 | i | .84 | .84 | *F*(3,1016)=1795.64, *p*<.001 |
|  | IS_T1 | .00 (.02) | [-.04, .04] | .910 |  |  |  |  |
|  | T1 z-BMI | .92 (.02) | [.88, .95] | .001 |  |  |  |  |
| 2 | GENDER | .00 (.01) | [-.02, .03] | .987 |  | .84 | .00 | *F*(1,1015)=.95, *p*=.331 |
|  | IS_T1 | .00 (.02) | [-.04, .04] | .988 |  |  |  |  |
|  | T1 z-BMI | .92 (.02) | [.88, .95] | .001 |  |  |  |  |
|  | T1_Lon | .01 (.01) | [-.01, .04] | .433 |  |  |  |  |
| 3 | GENDER | .00 (.01) | [-.02, .03] | .982 |  | .84 | .00 | *F*(1,1014)=.47, *p*=.492 |
|  | IS_T1 | .00 (.02) | [-.05, .04] | .956 |  |  |  |  |
|  | T1 z-BMI | .92 (.02) | [.88, .95] | .001 |  |  |  |  |
|  | T1_Lon | .02 (.02) | [-.02, .05] | .280 |  |  |  |  |
|  | T1_Lon^2^ | -.01 (.01) | [-.02, .02] | .625 |  |  |  |  |
| 4 | GENDER | .00 (.01) | [-.02, .03] | .979 |  | .84 | .00 | *F*(1,1013)=.00, *p*=.979 |
|  | IS_T1 | .00 (.02) | [-.04, .04] | .947 |  |  |  |  |
|  | T1 z-BMI | .92 (.02) | [.88, .95] | .001 |  |  |  |  |
|  | T1_Lon | .02 (.02) | [-.02, .05] | .276 |  |  |  |  |
|  | T1_Lon^2^ | -.01 (.01) | [-.02, .02] | .624 |  |  |  |  |
|  | T1_LonxGender | .00 (.01) | [-.01, .01] | .986 |  |  |  |  |
| 5 | GENDER | -.01 (.01) | [-.04, .02] | .656 |  | .84 | .00 | *F*(1,1012)=1.04, *p*=.308 |
|  | IS_T1 | .00(.02) | [-.05, .04] | .926 |  |  |  |  |
|  | T1 z-BMI | .92 (.02) | [.88, .95] | .001 |  |  |  |  |
|  | T1_Lon | .02 (.02) | [-.01, .05] | .257 |  |  |  |  |
|  | T1_Lon^2^ | -.01 (.01) | [-.02, .01] | .576 |  |  |  |  |
|  | T1_LonxGender | .00 (.01) | [-.01, .01] | .974 |  |  |  |  |
|  | T1_Lon^2^xGender | .00 (.01) | [-.01, .02] | .494 |  |  |  |  |

*Note.* T1=10 years old, T2=12 years old; z-BMI created using respondents’ BMI, self-reported age, Gender, and the external reference sample from WHO (Cole et al., 2000; de Onis et al., 2007); Lon= mean centred loneliness score; IS = Income Sufficiency; Bootstrap results are based on 1000 bootstrap samples*; Model:* *F* (7,1012)=768.74, *p*<.001

**Table S5.** Bootstrapped Hierarchical Regression Analysis: T1 Loneliness as a Predictor of Dependent Variable T3 z-BMI including quadratic effects and interactions.

|  | | Coefficients | | |  | Variance explained at each step | | |
| --- | --- | --- | --- | --- | --- | --- | --- | --- |
| Predictors | | B (SE) | 95% CI | *p* |  | R2 | ∆R2 | *F* Change |
| 1 | GENDER | .00 (.01) | [-.03, .03] | .870 | i | .79 | .79 | *F*(3,1016)=1261.25, *p*<.001 |
|  | IS_T1 | -.02 (.02) | [-.07, .03] | .492 |  |  |  |  |
|  | T1 z-BMI | .89 (.02) | [.85, .93] | .001 |  |  |  |  |
| 2 | GENDER | .00 (.01) | [-.03, .03] | .963 |  | .79 | .00 | *F*(1,1015)=5.26, *p*=.022 |
|  | IS_T1 | -.01 (.03) | [-.06, .04] | .643 |  |  |  |  |
|  | T1 z-BMI | .89 (.02) | [.85, .93] | .001 |  |  |  |  |
|  | T1_Lon | .03 (.01) | [.00, .06] | .060 |  |  |  |  |
| 3 | GENDER | .00 (.01) | [-.03, .03] | .933 |  | .79 | .00 | *F*(1,1014)=.35, *p*=.555 |
|  | IS_T1 | -.01 (.03) | [-.06, .04] | .611 |  |  |  |  |
|  | T1 z-BMI | .89 (.02) | [.85, .93] | .001 |  |  |  |  |
|  | T1_Lon | .04 (.02) | [-.01, .08] | .101 |  |  |  |  |
|  | T1_Lon^2^ | .00 (.01) | [-.02, .02] | .651 |  |  |  |  |
| 4 | GENDER | .00 (.01) | [-.03, .03] | .919 |  | .79 | .00 | *F*(1,1013)=1.66, *p*=.198 |
|  | IS_T1 | -.01 (.03) | [-.06, .04] | .585 |  |  |  |  |
|  | T1 z-BMI | .89 (.02) | [.85, .93] | .001 |  |  |  |  |
|  | T1_Lon | .04 (.02) | [-.01, .08] | .094 |  |  |  |  |
|  | T1_Lon^2^ | -.01 (.01) | [-.02, .02] | .633 |  |  |  |  |
|  | T1_LonxGender | .01 (.01) | [-.01, .02] | .313 |  |  |  |  |
| 5 | GENDER | -.01 (.02) | [-.05, .02] | .569 |  | .79 | .00 | *F*(1,1012)=1.32, *p*=.251 |
|  | IS_T1 | -.01 (.03) | [-.06, .04] | .574 |  |  |  |  |
|  | T1 z-BMI | .89 (.02) | [.86, .93] | .001 |  |  |  |  |
|  | T1_Lon | .04 (.02) | [-.01, .08] | .085 |  |  |  |  |
|  | T1_Lon^2^ | -.01 (.01) | [-.03, .02] | .577 |  |  |  |  |
|  | T1_LonxGender | .01 (.01) | [-.01, .02] | .322 |  |  |  |  |
|  | T1_Lon^2^xGender | .01 (.01) | [-.01, .02] | .415 |  |  |  |  |

*Note.* T1=10 years old; T3=13 years old; z-BMI created using respondents’ BMI, self-reported age, Gender, and the external reference sample from WHO (Cole et al., 2000; de Onis et al., 2007); Lon= mean centred loneliness score; IS = Income Sufficiency; Bootstrap results are based on 1000 bootstrap samples*; Model:* *F* (7,1012)=544.20, *p*<.001

**Table S6.** Bootstrapped Hierarchical Regression Analysis: T2 Loneliness (Lon) as a Predictor of Dependent Variable T3 z-BMI including quadratic effects and interactions.

|  | | Coefficients | | |  | Variance explained at each step | | |
| --- | --- | --- | --- | --- | --- | --- | --- | --- |
| Predictors | | B (SE) | 95% CI | *p* |  | R2 | ∆R2 | *F* Change |
| 1 | GENDER | .00 (.01) | [-.02, .02] | .951 | i | .89 | .89 | *F*(3,1030)=2827.98, *p*<.001 |
|  | IS_T2 | .01 (.02) | [-.02, .04] | .529 |  |  |  |  |
|  | T2 z-BMI | .95 (.02) | [.92, .97] | .001 |  |  |  |  |
| 2 | GENDER | .00 (.01) | [-.02, .02] | .951 |  | .89 | .00 | *F*(1,1029)=1.99, *p*=.158 |
|  | IS_T2 | .01 (.02) | [-.02, .04] | .462 |  |  |  |  |
|  | T2 z-BMI | .94 (.02) | [.91, .97] | .001 |  |  |  |  |
|  | T2_Lon | .01 (.01) | [-.01, .03] | .253 |  |  |  |  |
| 3 | GENDER | .00 (.01) | [-.02, .02] | .952 |  | .89 | .00 | *F*(1,1028)=6.84, *p*=.009 |
|  | IS_T2 | .01 (.02) | [-.02, .04] | .516 |  |  |  |  |
|  | T2 z-BMI | .95 (.02) | [.92, .97] | .001 |  |  |  |  |
|  | T2_Lon | -.02 (.02) | [-.04, .02] | .390 |  |  |  |  |
|  | T2_Lon^2^ | .02 (.01) | [.00, .03] | .083 |  |  |  |  |
| 4 | GENDER | .00 (.01) | [-.02, .02] | .954 |  | .89 | .00 | *F*(1,1027)=.48, *p*=.490 |
|  | IS_T2 | .01 (.02) | [-.02, .04] | .502 |  |  |  |  |
|  | T2 z-BMI | .95 (.02) | [.92, .97] | .001 |  |  |  |  |
|  | T2_Lon | -.01 (.02) | [-.04, .02] | .423 |  |  |  |  |
|  | T2_Lon^2^ | .02 (.01) | [.00, .03] | .085 |  |  |  |  |
|  | T2_LonxGender | .01 (.01) | [-.02, .03] | .586 |  |  |  |  |
| 5 | GENDER | -.03 (.01) | [-.05, .00] | .035 |  | .89 | .00 | *F*(1,1026)=11.37, *p*=.001 |
|  | IS_T2 | .01 (.02) | [-.02, .04] | .528 |  |  |  |  |
|  | T2 z-BMI | .94 (.02) | [.91, .97] | .001 |  |  |  |  |
|  | T2_Lon | -.01 (.02) | [-.04, .02] | .435 |  |  |  |  |
|  | T2_Lon^2^ | .01 (.01) | [.00, .02] | .069 |  |  |  |  |
|  | T2_LonxGender | -.03 (.02) | [-.05, .01] | .044 |  |  |  |  |
|  | T2_Lon^2^xGender | .02 (.01) | [.00, .03] | .007 |  |  |  |  |

*Note.* T2=12 years old; T3=13 years old; z-BMI created using respondents’ BMI, self-reported age, Gender, and the external reference sample from WHO (Cole et al., 2000; de Onis et al., 2007); Lon= mean centred loneliness score; IS = Income Sufficiency; Bootstrap results are based on 1000 bootstrap samples*; Model:* *F*(7,1026)=1234.70, *p*<.001.
